# An exploration of factors affecting the quality of life of women with primary ovarian insufficiency: a qualitative study

**DOI:** 10.1186/s12905-020-01029-y

**Published:** 2020-08-05

**Authors:** Samira Golezar, Zohreh Keshavarz, Fahime Ramezani Tehrani, Abbas Ebadi

**Affiliations:** 1 Department of Midwifery, Faculty of Nursing and Midwifery, Kermanshah University of Medical Sciences, Kermanshah, Iran; 2 Department of Midwifery and Reproductive Health, School of Nursing and Midwifery, Shahid Beheshti University of Medical Sciences, Tehran, Iran; 3 Reproductive Endocrinology Research Center, Research Institute for Endocrine Sciences, Shahid Beheshti University of Medical Sciences, Tehran, Iran; 4 Behavioral Sciences Research Center, Life Style Institute, Baqiyatallah University of Medical Sciences, Tehran, Iran

**Keywords:** Primary ovarian insufficiency, Quality of life, Qualitative research

## Abstract

**Background:**

Menopause before the age of 40 years is known as primary ovarian insufficiency (POI). Besides physical effects, being diagnosed with this disorder adversely affects the psychological health and quality of life (QOL). The present study aimed at shedding light on the factors affecting the QOL of women with POI.

**Methods:**

The present study is a qualitative one. The data were collected using semi-structured in-depth interviews with 16 women having POI, selected purposively. Data rigor was ensured using Lincoln and Guba’s criteria. The recorded data were transcribed verbatim and then analyzed constantly at the same time as gathering the data using conventional content analysis.

**Results:**

Three themes emerged regarding the QOL of women with POI, i.e. disease effect (physical and psychological effects), distorted self-concept (threatened identity and disease stigma), and hormone replacement therapy effect (positive and negative physical/psychological effects).

**Conclusions:**

Due to the profound effects of the disease on different aspects of the biopsychosocial health of women with POI, a multifaceted health care approach is recommended to improve their QOL.

## Background

Menopause before the age of 40 is called POI, mentioned as a premature ovarian failure or premature menopause, identified by oligo/amenorrhea for at least 4 months, and an elevated FSH level > 25 IU/I on two occasions 4 weeks apart [1, 2]. In a meta-analysis, the global prevalence of POI wad reported as 3.7% [3]. Also, another meta-analysis of 9 cohort studies reported a prevalence of 2% in women in natural menopause [4]. In a study on the women in natural menopause in the city of Zabol, Iran, a POI prevalence of 5.9% was reported [5]. A national population-based survey of 4898 Iranian women reported 3.2% of the participants as experiencing POI [6].

POI occurs either spontaneously or as a result of medical interventions, including chemotherapy or bilateral oophorectomy [7]. Symptoms associated with deficiency of estrogen, irregular menstruations, and infertility impairment are among POI presentations [8].

Spontaneous POI exposes women to an accelerated risk of chronic sequelae such as osteoporosis and fractures, overall cardiovascular disease, stroke, Type 2 diabetes, and total mortality [8, 9].

Besides physical effects, being diagnosed with POI adversely affects the psychological health and QOL of women [4, 10]. Women having POI reportedly have a high level of depression, lose self-esteem, and experience adverse effects on their sexuality [11]. Some studies have shown that the QOL for women with POI is lower compared to that of the control group [12–14].

Although the QOL plays a vital role in women’s health, to the knowledge of the authors, no qualitative studies have been carried out throughout the world focusing on the QOL of women with POI.

There is a direct relationship between health and the QOL, hence there is a strong need to assess how a patient perceives the QOL affecting, as a subjective entity, her well-being which, according to recent studies, will in turn influence morbidity and mortality as an objective one [12]. Moreover, the QOL is, by definition, how an individual perceives their status within their habitus (i.e. cultural context, set of norms and values, expectations, and interests) [15]. With regard to what was mentioned above, the present study aimed at shedding light on the factors affecting the QOL of women with POI with reference to the cultural context of Iranian society.

## Methods

The present study is the qualitative phase of a sequential qualitative-quantitative exploratory study on the QOL experiences of POI women.

### Participant recruitment

The study population was women with POI referred to the gynecology clinic of the Research Institute of Endocrine Sciences of Shahid Beheshti University of Medical Sciences, Tehran, Iran, who met the inclusion criteria.

The inclusion criteria were women with spontaneous POI based on the diagnostic criteria, disorder duration of at least 1 year, being oriented and alert, being of Iranian nationality and Farsi speaking, and not having a history of psychological or disabling chronic diseases. The POI diagnosis criteria included: experiencing amenorrhea lasting at least 4 months before the age of 40 accompanied with two FSH serum levels of more than 25 mIU/ml, and tested with at least a one-month interval [1] which was subsequently confirmed by a gynecologist (FRT). Women were contacted by phone and if they were inclined to participate in the study, the time and place of the interviews were arranged. Purposive sampling was performed with a maximum variation of sampling in terms of age, education, marital status, and parity and continued until data saturation i.e. until no new themes arise from further data collection [16].

### Interviews

In-depth semi-structured interviews were used to collect the data. First, the participants were asked about their personal information including education, occupation, menarche age, duration of the disease, marital status, family history of POI, having children, and type of pregnancy. Afterward, general and open-ended questions were asked using the interview guide which was designed based on pilot interviews with 3 participants and used after being reviewed by the research team. The interview guide developed for this study is provided as the Additional File 1. The sequence of questions was not the same for all participants and depended on the interview process. The subsequent questions were asked for clarification purposes based on the women’s answers about POI. Also, probing questions were used such as: “Would you explain in more detail?” and “What do you mean?”. The interview setting was, depending on the participants’ preferences, either a private room in the clinic or at their home. The main researcher for the study (SG) conducted the interviews. Prior to the interviews, the purpose of the research was explained to the participants, they were informed that the interviews would be recorded, and they were also assured of the confidentiality of their personal information. The interviews began in July 2017 and ended in January 2018, lasting between 40 and 105 min (mean 50 min).

### Ethical considerations

Ethical approval to conduct this study (IR.SBMU.PHNM.1395.529) was granted by the Ethics Committee of the School of Nursing and Midwifery, Shahid Beheshti University of Medical Sciences, Tehran, Iran. Prior to the interviews, the purpose of the research was explained to the participants, they were informed that the interviews would be recorded, and they were also assured of the confidentiality of their personal information. Informed consent was obtained from all individual participants included in the study.

### Data analyses

The gathered data were analyzed using the content analysis method with a conventional approach [17]. Immediately after each interview, the recorded data were transcribed verbatim. To get immersed in the data, the main researcher of the study read the transcriptions repeatedly while checking them against the recordings.

The data analysis was performed in six stages [16]: (1) getting familiar with the data; (2) generating the initial codes; (3) searching for the themes; (4) reviewing the themes (5) defining and naming the themes; and (6) producing a report. To do so, the initial codes were extracted from the meaning units (participants’ quotations). Then, the main codes, which were more abstract, were named based on the similaritis of these codes and subsumed, in terms of their common characteristics, under congruent subcategories. Then, each set of related sub categories were put under a main category. Finally, the themes emerged out of the main categories conveying a common concept.

### Data quality

Rigor and conformability of the data were ensured using the four criteria proposed by Lincoln and Guba [18]. The credibility of the data was confirmed through prolonged engagement with the data for 1 year and giving reflective commentaries; then to address further rigor, the data was member-checked. Afterward, the codes and extracted categories were peer-checked to reach consensus. The three data collection methods (in-depth individual interview, observation, and note-taking) were triangulated and the time-integration method was applied. To establish transferability, samples with the highest level of knowledge were chosen, and maximum variation sampling was implemented.

Conformability was ensured using an external check. To confirm dependability, in addition to creating an audit trail, the researcher recoded the same interview transcriptions with an interval of a few days and compared the outcomes. Also, the transcriptions of the initial interviews were recoded by two colleagues with Ph.D.s in reproductive health. Ultimately, a 95% consensus was achieved through an external check, peer-check, and dependability items.

## Results

In this study, 16 women with POI, aged between 28 and 47 years, and a POI duration of 2–15 years were interviewed. The demographic characteristics of the participants are summarized in Table 1.

**Table 1:** Demographic and reproductive characteristics of the participants

Characteristics	Mean (range)
**Age (year)**	36.68(28–47)
**Menarche Age (year)**	12.68 (9–17)
**Disease Duration (year)**	6 (2–15)
**Characteristics**	**Number (percent)**
**Education**	
Primary	1 (6.25%)
Diploma	4 (25%)
Associate’s Degree	1 (6.25%)
Bachelor Degree	6 (37.5%)
Master’s Degree	4 (25%)
**Occupation**	
Housewife	7 (43.75%)
Employed	7 (43.75%)
Student	2 (12.5%)
**Marital status**	
Single	4 (25%)
Married	10 (62.5%)
Divorced	2 (12.5%)
**Have Children**	
Yes	6 (37.5%)
No	10 (62.5%)
**Type of Pregnancy**	
Natural	5 (71.4%)
Donor Egg	2 (28.57%)
**POI Family History**	
Yes	7 (43.75%)
No	9 (56.25%)

After content analysis of the interviews with a focus on the factors influencing the QOL of women with POI, three themes emerged (disease effect, distorted self-concept, and hormone replacement therapy effect), explained as follows (see Table 2).

**Table 2:** The themes, main categories, and sub-categories of the POI women’s QOL experiences

Theme	Main Category	Sub-Category
**Disease Effect**	Physical Effects	Menstrual Disorders Vasomotor Disorders Fertility Disorders Bone complications Mucocutaneous Complications Sexual Function Disorders General Health Disorders
	Psychological Effects	Consternation Grief Rage Moodiness Stress Negative Feelings
**Distorted Self-Concept**	Threatened Identity	Threatened Femininity Threatened Maternal Role Distorted Body Image
	Disease Stigma	Concealment Being Judged
**HRT Effect**	Positive/ Desirable Effects	Psychological Physical
	Negative/ Undesirable Effects	

### Disease effect

Consisting of two main categories i.e. physical and psychological, the theme is defined here as the direct negative influences POI exerts on the various aspects of a woman’s health, taking a toll on her QOL.

#### Most participants experienced menstrual, vasomotor, sexual function, and general health disorders, as well as bone and mucocutaneous complications during POI

The very first complaints of POI in these women were menstrual disorders including menstrual irregularities, oligomenorrhea, and in some cases, metrorrhagia, occurring 6 months to 6 years prior to the final diagnosis. Two of them had primary amenorrhea. Many of the women with POI complained about vasomotor disorders such as hot flushes, night sweats, and heat intolerance.

*“I can’t bear heat or thirst. It’s been more intense in the last six months. When I begin to fall asleep at night, suddenly the hot flushes come about. It’s as if I am burning from the inside. I can’t go to sleep anymore when this happens, you know, because of the rapid heartbeat and all the sweating” (20's-30's, disease duration: 2 years).*

Fertility disorders were also caused by POI. The women inclined to have a baby complained about infertility. Those resorting to assisted reproductive technology (ART) mentioned donor egg pregnancy, abortion, and in-vitro-fertilization (IVF) as factors reducing their QOL and putting a long-lasting strain on them.

As for bone complications, two of the most commonly experienced issues were joint pain and osteoporosis. Also, a few of the women complained about post-menopausal tooth pain and sensitivity.

*“I have osteoporosis and severe joint pain. I feel pain deep in my bones. I can’t take long walks” (40's-50's, disease duration: 13 years).*

The mucocutaneous complications were reported by almost all participants as occurring in the form of vaginal or skin dryness; vaginal itchiness and tightness; and reported by a few, there were falling hair and wrinkled skin.

*“Dryness and itchiness drive me crazy. Sometimes I scratch myself to bleeding. You wouldn’t want to know how awful it is when at work, it hurts so much that I like to chop it off.” (40's-50's, disease duration: 3 years)*

The afflicted women would mainly experience sexual function disorders due to ovarian hypofunction. They experienced dyspareunia, reduced sex drive, and anorgasmia.

*“The disease has affected my sex life. I don’t feel like having sex at all. Last time I had sex, it was so, so painful and hurt a lot. I just tried to cope up with it and make as if it wasn’t there but I could never have an orgasm.” (30's-40's, disease duration: 6 years)*

Despite these disorders, most women expressed that their frequency of having sex remained unchanged.

*“Now that I am disabled and not a perfect woman anymore, I want to manage it and have a kind of normal sex life. I don’t want my husband to feel deprived. I would like him to have a normal sex life.” (30's-40's, disease duration: 3 years).*

Many women were in good health, however, some of them reported conditions like weariness, loss of physical strength, and sleep disorders.

*“I feel as if I had become heavier … when you don’t get period, you’re down … you’re not that agile anymore. You’re bored and not fresh.” (30's-40's, disease duration: 4 years).*

#### Based on the analysis of the interviews, the participant’s experiences of the POI psychological effects included shock, grief, rage, moodiness, stress and anxiety, as well as negative feelings, all of which influenced the participants’ QOL in different ways

According to a majority of the participants, being diagnosed with the disease was shocking and unbelievable:

*“It came to me like a blow. I felt awful. I was shocked and frustrated. I was in shock for some time.” (30's-40's, disease duration: 3 years).*

The women experienced grief for quite a long time after being diagnosed with the disease. They were concerned about the complications of the disease (e.g. infertility, sexual problems, and osteoporosis), and couldn’t easily talk or even think about it:

*“I am so disappointed. Everyone dreams of having a baby. I cry when I’m alone and think of it … , that I cannot experience it naturally. When others are talking about children or I see a little child, I get even more disappointed” (30's-40's, primary amenorrhea).*

There were many cases of becoming aggressive, agitated and losing control over anger associated with POI, reported by the interviewees. The conditions associated with fits of moodiness in these women included rage, mood swings, impatience, and, as reported by some of them, introversion.

*“Before receiving the treatments, I experienced all those changes of temperaments, you know, you suddenly get happy and then, for no reason you start to cry. I was beginning to feel helpless because it hurt so much.” (30's-40's, disease duration: 5 years)*

Also, there was a large number of these women complaining about stress-related problems such as anxiety, tension, and lack of concentration while before having POI, they never had such an experience at this level.

*“I am anxious; I very much like to read a book but I just can’t seem to be able to finish it because of all the anxiety I have. I am trying to tell you that I lack concentration.” (30's-40's, disease duration: 4 years)*

The leading causes of POI women’s anxiety were as follows: losing health, having children, and getting married. Other causes had roots in physical effects of the disease, its economic burden, fear of future incidence of the possible related complications, and its turning into a chronic disease:

*“I’m worried about getting married. I’m afraid there will be no Mr. Right accepting me as a girl getting to menopause at an early age who cannot give him a baby”. (30's-40's, disease duration: 4 years)*

Subsequent to POI-induced infertility as well as menopausal complications, many women experienced negative feelings including hopelessness, emptiness, being cursed, and unhappiness:

*“I feel empty for being infertile. I can’t enjoy real happiness … why should I be that unlucky? My peers get periods and are healthier than me.” (40's-50's, disease duration: 10 years)*

### Distorted self-concept

The analysis of the experiences of women with POI yielded factors such as threatened identity and disease stigma, distorting their self-concept and adversely affecting their QOL.

#### Threatened femininity and maternal role, as well as a distorted body image, formed the subcategories of “threatened identity”

Femininity was threatened by amenorrhea and followingly, infertility; as a consequence, women would experience feelings like deficiency, losing self-confidence, femininity defect, being different from other women, and embarrassment. Some women even resisted entering a relationship with the opposite sex:

*“I feel disabled; I am not an all-around woman anymore. Compared to normal women, I lack something. It’s as if I’m weaker than other women. I feel I am sterilized.” (30's-40's, disease duration: 3 years)*

The identity, and as a result, the maternal role of POI women who wanted to have children were threatened since they couldn’t have the natural experience of a genetic mother. Their main concerns turned out to be: forced acceptance of a donor egg, the donor egg child’s lack of resemblance to them, not being accepted as a mother by the child, and the egg donor claiming the baby.

*“I accepted the donor egg to save my marriage, but there are some things to worry about. What if the child leaves me because I am not his/her genetic mother? What if the egg donor shows up and claims the baby one day or another?” (30's-40's, initial amenorrhea)*

POI had caused an undesirable self-image in women making them feel aged, ‘withered’, disabled during intercourse, and with deteriorated self-confidence as a result of breast sagging and poor fitness. One of the women explains:

*“A woman with POI is like a flower withered before blooming. I feel so old; it is as if I am too old for my age. I’m not youthful anymore, I’m withered.” (30's-40's, disease duration: 4 years).*

Another interviewee states that:

*“I feel like old women when I have to take calcium pills at this age to maintain strong bones.” (30's-40's, disease duration: 5 years)*

#### The psychosocial aspects of POI created stigmas for women; besides, infertility and menopause-related social feedbacks distorted their self-concept and affected their quality of social life in general. Experienced by these women, concealment and being judged were two subcategories of this aspect

Most of the participants resorted to concealment due to the disease stigma. The afflicted women and the donor egg receivers intended to hide the disease and the donor egg from others. Also, some women reported feelings of isolation after having POI.

*“One of the problems I have with the diseases is that I have to hide it because I don’t like anyone to find out about it. You need to make believe that you are fine while having it with you.” (20's-30's, disease duration: 2 years)*

The interviewees reported that they suffered consequences of the disease stigma such as being judged, being labeled, being blamed, looking pathetic, people’s scornful look, and the bad reputation of the disease.

*“I kind of feel like it’s becoming a drawback for me and my husband is using it against me. The moment something comes up, he brings it up and then it’s me with egg on my face.” (30's-40's, disease duration: 6 years)*

### Hormone replacement therapy effect

As a symptomatic therapy influencing the QOL, hormone replacement therapy (HRT) was administered to POI women. Two main categories emerged out of the participants’ experiences, i.e. positive or desirable effects, and negative or undesirable ones.

#### HRT assisted in the regulation of the women’s menstrual cycles producing positive psychological effects. Receiving HRT, they reported effects like stress reduction, depression improvement, mood swings improvement, and elimination of unwanted thoughts. The positive physical effects were an improvement in hot flushes and vaginal dryness

*“I take medicine to regulate my menstruation. My period is regular now and I have no hot flushes. I don’t think of how it affects my health” (40's-50's, disease duration: 3 years)*

#### Getting nervous and feeling tired of taking hormones on a daily basis for a long time were among the HRT-related psychological experiences of the women with POI. The main causes of the physical complaints were weight gain, weight fluctuations, and poor fitness. Some women also reported cases of nausea, migraine, falling or thinning hair, and acne

*“I have gained lots of weight since I took the medicines. Now, I have stopped using them by myself. I couldn’t swallow the pills. I was fed up with them.” (30's-40's, disease duration: 5 years)*

## Discussion

The present study adopted a qualitative approach to the analysis of the QOL experiences of Iranian women with POI. The results showed that several interrelated factors could affect women’s QOL.

Here, the “disease effect” is taken as the understanding and experience women have of the physical and psychological effects of POI on their QOL. The decrease in ovarian hormones triggers a set of symptoms capable of affecting the women’s QOL. Studies have shown that menopause was accompanied by a reduction in the QOL of women due to its physical and psychological effects [12, 19].

The interviewees mentioned menstrual irregularities as the earliest symptom that engaged them for months before the cycles stopped permanently. Similarly, Alzubaidi et al. reported menstrual irregularities as the most common early symptom among women with POI, lasting 3 to 5 months from the appearance of the symptoms to the final diagnosis [20]. However, as a result of the sudden cessation of mensuration, cases of induced menopause did not experience any such irregularities [21].

In line with the results of other studies [20, 22], our study indicated that a common complaint among the participants of the present study was hot flushes disrupting their normal life and making them resort to HRT or herbal medicine.

In the current study, the sexual disorder of the majority of the interviewees led to reducing the quality of their sexual life. Sexual dysfunction in women could negatively affect QOL [23]. In line with our study, Orshan et al. mention low sex drive, vaginal dryness, and dyspareunia as commonly experienced by these women [24]. Singer et al. also report loss of sexual desire and vaginal dryness as the most prominent problems of POI women in addition to infertility [25]. A quantitative study revealed that POI women experienced more pain and were less sexually aroused and lubricated, which caused them to be less satisfied with their sexual life than the control group [14].

Losing fertility could negatively affect the POI women’s interest in having sex to the extent that they regarded it as useless [26]. However, for the most part, to meet their husbands’ sexual needs, for the fear of their husbands starting an extra-marital relationship, and to a lesser extent, to compensate for the perceived deficit, women in the present study tried not to let their lack of sexual desire hamper their sexual relationship and its frequency.

Osteoporosis and joint pain were also among experiences putting restrictions on women’s activities in the current study; although a long-term side effect of POI, the two were not correlated with the disease duration. This could be related to race, nutrition, physical activity, and lifestyle differences [27]. A study reported 80% of POI women complaining about joint pain [25].

In accordance with other studies [22, 24, 25], there were other physical effects reported by the participants including feeling fatigue, sleeping disorders, loss of physical strength, falling hair, and weight gain.

Losing fertility came as a great shock to the women in the study. Furthermore, they experienced a set of psychological symptoms due to being subject to health risks or hormone disorders following POI. Other studies have reported common feelings of shock, confusion, rage, sorrow, loss, depression, excitability, anxiety, and emptiness among women with POI [22, 24, 28]. Participants in Orshan et al.’s study described the moment they were diagnosed with POI as the moment of death [24] and those in Groff et al.’s study, described it as devastative [28]. In a study, women were reported as experiencing deep sorrow for losing fertility and future opportunities [29].

In the present study, “distorted self-concept” included threatened identity and disease stigma. It has been shown that self-concept and QOL are directly related [30]. Pasquali showed that as a result of POI, the women’s self-concept changes from a fertile menstruating woman to an infertile and post-menopausal one. Also, it was revealed that threatened femininity and maternal role, along with a distorted body image threatened POI women’s identity [22].

Losing fertility made women feel deficient and older than their peers. Part of Iranian women’s identity is their maternal role and childbearing, giving them power both in the family and society. So, losing fertility affects all the elements of a woman’s identity (personal, social, and family) [31]. In Orshan et al.’s study, women felt like they were robbed of something [24]. In Groff et al.’s study, the participants explained that POI had adversely affected their body image and sense of self, i.e. they felt as if they were less feminine and more aged [28]. In Pasquali’s study, women felt less feminine and attractive for losing fertility and bodily changes [21].

Women in the present study were grappling with POI-induced stigmas so that hiding the disease became a major obsession to them. The bad reputation of the disease and lack of knowledge and understanding on the part of others comparing them to old women made the participants conceal the disease and opt for isolation.

From society’s view and even in the medical discourse, menopause is synonymous with oldness [29]. Infertility not only affected women’s self-concept but it also affected the perception of others toward infertile women; also, because childbearing was considered to be a norm, being infertile turned into a social stigma [31]. For the same reason, women in the present study would tend to hide their being infertile or receiving a donor egg.

In line with findings of the present work, the results of other studies on lived experiences of infertile Iranian women showed that due to the negative reactions, they intended to terminate their relations with their spouse’s relatives or hide their problem [31, 32]. Similarly, In Pasquali’s study women never said a word about POI to their mothers or relatives [21]. In Boughton’s study, women were afraid to let others know about their menopause because they resented being described with cliché attributes such as aged, disabled, not attractive, and infertile [29].

In the present study, “HRT effect” included physical and psychological advantages, despite some minor negative effects experienced by the participants. Induction and regulation of menstrual cycles were of prime importance to the participants and they found it solacing to feel like their non-menopausal peers. However, these women complained about being tired of long-term consumption of medicine and weight gain. In Singer et al.’s study, the long-term consumption of the medicine was reported to be a source of problem to the POI women [25]. In Orshan et al.’s study, using HRT turned out to be embarrassing to some women and induced a sense of old age in them [24].

Some studies have shown that HRT could lead to the improvement of QOL in menopausal women [33]. HRT risks and advantages are not well-documented for young women, however, it is strongly recommended to young patients under 50 suffering symptoms of menopause if there are no contraindications. This is done with the aim of minimizing the probability of long-term sequelae and optimizing the QOL [8, 11].

The limitations of the study were that participant selection was done purposively and from large urban areas; moreover, only the experiences of patients who consented to participate were included. These might hamper the generalizability of the results. However, this qualitative study contributes greatly to the existing literature since it’s the first qualitative research carried out on the QOL of POI women around the world. Further studies will aid in the elaboration of the QOL of these women in other parts of the world.

## Conclusion

It is concluded that POI affects different aspects of a woman’s life as well as her health and in addition to exerting physical and psychological effects, it distorts women’s self-concept. Also, it was revealed that HRT affects POI women’s QOL both positively and negatively. Due to the profound effects of the disease on different biopsychosocial aspects of women, its hidden complications require ample attention on the part of health providers to enhance their QOL through multifaceted health services. The findings of the present study could serve as a stepping stone to the development of a POI women’s QOL questionnaire.

## Supplementary information

**Additional file 1.**

## Data Availability

Data sharing is not applicable to this article as all transcripts and recordings were deleted permanently following data analysis to protect the identity and privacy of the participants.

## Abbreviations

POI: Primary ovarian insufficiency
QOL: Quality of life
HRT: Hormone replacement therapy
ART: Assisted reproductive technology
IVF: In-vitro-fertilization

## References

[1] Webber L, Davies M, Anderson R, Bartlett J, Braat D, Cartwrigh B, et al. ESHRE guideline: management of women with premature ovarian insufficiency. Hum Reprod. 2016. 10.1093/humrep/dew027. 10.1093/humrep/dew027 27008889

[2] Jin M, Yu Y, Huang H. An update on primary ovarian insufficiency. Sci Chin Life Sci. 2012. 10.1007/s11427-012-4355-2. 10.1007/s11427-012-4355-2 22932883

[3] Golezar S, Ramezani Tehrani F, Khazaei S, Ebadi A, Keshavarz Z. The global prevalence of primary ovarian insufficiency and early menopause: a metaanalysis. Climacteric. 2019. 10.1080/13697137.2019.1574738. 10.1080/13697137.2019.1574738 30829083

[4] Mishra GD, Pandeya N, Dobson AJ, Chung HF, Anderson D, Kuh D, et al. Early menarche, nulliparity and the risk for premature and early natural menopause. Hum Reprod. 2017. 10.1093/humrep/dew350. 10.1093/humrep/dew350 PMC5850221 28119483

[5] Delavar MA, Hajiahmadi M. Age at menopause and measuring symptoms at midlife in a community in Babol, Iran. Menopause. 2011. 10.1097/gme.0b013e31821a7a3a. 10.1097/gme.0b013e31821a7a3a 21792079

[6] Parsaeian M, Pouraram H, Djazayery A, Abdollahi Z, Dorosty A, Jalali M, et al. An explanation for variation in age at menopause in developing countries based on the second national integrated micronutrient survey in iran. Arch Iran Med. 2017; doi:0172006/AIM.008. 28646845

[7] Shuster LT, Rhodes DJ, Gostout BS, Grossardt BR, Rocca WA. Premature menopause or early menopause: long-term health consequences. Maturitas. 2010. 10.1016/j.maturitas.2009.08.003. 10.1016/j.maturitas.2009.08.003 PMC2815011 19733988

[8] Hewlett M, Mahalingaiah S. Update on primary ovarian insufficiency. Curr Opin Endocrinol Diabet Obes. 2015. 10.1097/MED.0000000000000206. 10.1097/MED.0000000000000206 PMC4883575 26512773

[9] Gong D, Sun J, Zhou Y, Zou C, Fan Y. Early age at natural menopause and risk of cardiovascular and all-cause mortality: a meta-analysis of prospective observational studies. Int J Cardiol. 2016. 10.1016/j.ijcard.2015.10.092. 10.1016/j.ijcard.2015.10.092 26512821

[10] Golezar S, Ramezani TF, Ebadi A, et al. Coping with primary ovarian insufficiency in iranian women: a qualitative study. J Isfahan Med School. 2019. 10.22122/jims.v37i529.11019.

[11] Goswami D, Conway GS. Premature ovarian failure. Horm Res. 2007:196–202. 10.1159/000102537. 10.1159/000102537 17495481

[12] Benetti-Pinto CL, De Almeida DM, Makuch MY. Quality of life in women with premature ovarian failure. Gynecol Endocrinol. 2011. 10.3109/09513590.2010.520374. 10.3109/09513590.2010.520374 21214499

[13] Islam R, Cartwright R (2011). The impact of premature ovarian failure on quality of life. Hum Reprod.

[14] Van der Stege JG, Groen H, Van Zadelhoff SJ, Lambalk CB, Braat DD, van Kasteren YM, et al. Decreased androgen concentrations and diminished general and sexual well-being in women with premature ovarian failure. Menopause. 2008. 10.1097/gme.0b013e3180f6108c. 10.1097/gme.0b013e3180f6108c 18257141

[15] Group WHO. The World Health Organization quality of life assessment (WHOQOL): position paper from the World Health Organization. Soc Sci Med. 1995. 10.1016/0277-9536(95)00112-K. 10.1016/0277-9536(95)00112-k 8560308

[16] Speziale HS, Streubert HJ, Carpenter DR (2011). Qualitative research in nursing: advancing the humanistic imperative.

[17] Hsieh H-F, Shannon SE. Three approaches to qualitative content analysis. Qual Health Res. 2005. 10.1177/1049732305276687. 10.1177/1049732305276687 16204405

[18] Lincoln YS, Guba EG (1985). Naturalistic inquiry.

[19] Avis NE, Colvin A, Bromberger JT, et al. Change in health-related quality of life over the menopausal transition in a multiethnic cohort of middle-aged women: study of Women’s health across the nation (SWAN). Menopause. 2009. 10.1097/gme.0b013e3181a3cdaf. 10.1097/gme.0b013e3181a3cdaf PMC2743857 19436224

[20] Alzubaidi NH, Chapin HL, Vanderhoof VH, Calis KA, Nelson LM. Meeting the needs of young women with secondary amenorrhea and spontaneous premature ovarian failure. Obstet Gynecol. 2002. 10.1016/S0029-7844(02)01962-2. 10.1016/s0029-7844(02)01962-2 11978278

[21] Pasquali EA. The impact of premature menopause on women’s experience of self. J Holist Nurs. 1999. 10.1177/089801019901700404. 10.1177/089801019901700404 10818847

[22] Pasquali EA. Premature menopause and self-concept disjunctions: a case for crisis management. J Psychosoc Nurs Ment Health Serv. 2002. 10.3928/0279-3695-20020901-09. 10.3928/0279-3695-20020901-09 12235966

[23] Nazarpour S, Simbar M, Ramezani Tehrani F (2016). Relationship between sexual function and quality of life in post-menopausal women. J Mazandaran Univ Med Sci.

[24] Orshan SA, Furniss KK, Forst C, Santoro N. The lived experience of premature ovarian failure. J Obstet Gynecol Neonatal Nurs. 2001. 10.1111/j.1552-6909.2001.tb01536.x. 10.1111/j.1552-6909.2001.tb01536.x 11308110

[25] Singer D, Mann E, Hunter M, Pitkin J, Panay N. The silent grief: psychosocial aspects of premature ovarian failure. Climacteric. 2011. 10.3109/13697137.2011.571320. 10.3109/13697137.2011.571320 21762006

[26] Graziottin A, Basson R. Sexual dysfunction in women with premature menopause. Menopause. 2004. 10.1097/01.GME.0000139926.02689.A1. 10.1097/01.gme.0000139926.02689.a1 15543028

[27] Saadi H, Reed R, Carter A, Qazaq H, Al Suhaili A (2001). Bone density estimates and risk factors for osteoporosis in young women. East Mediterr Health J.

[28] Groff AA, Covington SN, Halverson LR, Fitzgerald OR, Vanderhoof V, Calis K, et al. Assessing the emotional needs of women with spontaneous premature ovarian failure. Fertil Steril. 2005. 10.1016/j.fertnstert.2004.11.067. 10.1016/j.fertnstert.2004.11.067 15950644

[29] Boughton MA. Premature menopause: multiple disruptions between the woman's biological body experience and her lived body. J Adv Nurs. 2002. 10.1046/j.1365-2648.2002.02114.x. 10.1046/j.1365-2648.2002.02114.x 11843980

[30] Safavi M, Samadi N, Mahmoodi M (2013). The relationship between self-concept and quality of life in patients with type 2 diabetes. Med Sci J Islam Azad Univ.

[31] Karimi M, Omani SR, Shirkavand A (2015). A qualitative study of the experiences of infertile woman in Iran. Payesh.

[32] Savadzadeh S, Madadzadeh N (2013). Explanation of emotional feelings of women with infertility: a qualitative study. J Ilam Univ Med Sci.

[33] Blumel JE, Castelo-Branco C, Binfa L, et al. Quality of life after the menopause: a population study. Maturitas. 2000. 10.1016/s0378-5122(99)00081-x. 10.1016/s0378-5122(99)00081-x 10687878

