# Supplementary material for: An exploration of factors affecting the quality of life of women with primary ovarian insufficiency: a qualitative study
Source: BMC Womens Health. 2020 Aug 5;20:163. doi: 10.1186/s12905-020-01029-y (PMC7405332; doi:10.1186/s12905-020-01029-y)
Supplement: Supplementary file 1 — Additional file 1. [file 12905_2020_1029_MOESM1_ESM.docx]

Interview guide

- What problems did you face since the disorder started?”

- How did POI influence (positively or negatively) your quality of life (physical/sexual/marital/psychological)?

- What are the effects of POI (positive/negative) on your relationships with those around you (spouse, family, colleagues)?

- What are the effects of POI on your feeling about health?

- What is your main concern about POI?

- Do you know people whose quality of life is better / worse than yours, despite having this disorder? What are their characteristics?

- What strategies have you used to cope with POI?
